# Prevalence of Hypertension and Adherence to Dietary Approaches to Stop Hypertension Diet Score in Childbearing Age Tunisian Women: A Cross-Sectional Study

**DOI:** 10.1155/2021/6686299

**Published:** 2021-11-05

**Authors:** Jalila El Ati, Radhouene Doggui, Houda Ben Gharbia, Myriam El Ati-Hellal

**Affiliations:** ^1^INNTA (National Institute of Nutrition and Food Technology), SURVEN (Nutrition Surveillance and Epidemiology in Tunisia) Research Laboratory, 11 Rue Jebel Lakhdar, Bab Saadoun, 1007 Tunis, Tunisia; ^2^IPEST (Preparatory Institute for Scientific and Technical Studies), Laboratory Materials Molecules and Applications, University of Carthage, P.B. 51, 2070 Tunis, Tunisia

## Abstract

**Background and Aims:**

The prevalence, awareness, and treatment of hypertension, along with their sociodemographic, anthropometric, and lifestyle associations, were evaluated in a cross-sectional survey of childbearing age Tunisian women. Adherence to the Dietary Approaches to Stop Hypertension diet score was also assessed for hypertensive versus nonhypertensive women.

**Methods and Results:**

A total of 1689 nonpregnant women, aged 20-49 years, were randomly sampled a regional (Greater Tunis), two-stage, stratified, cross-sectional cluster survey from March 2009 to January 2010. Data on medical history and sociodemographic characteristics were collected using a questionnaire. The average daily intake of energy and nutrients was computed using a specific Tunisian food composition database. The Dietary Approaches to Stop Hypertension diet score (0 to 10) was assessed by adding the individual scores (0 to 1) of ten nutrient components according to dietary guidelines. The overall prevalence of hypertension was 21.4%. Age, obesity, abdominal fat, parity, and family history were significantly associated with hypertension. The mean Dietary Approaches to Stop Hypertension accordance score was 4.93 for hypertensive women and 4.86 for nonhypertensive women (*P* = 0.0556). After adjustment for age, energy intake, and all nutritional covariates, no associations were observed between hypertension and Dietary Approaches to Stop Hypertension diet components.

**Conclusion:**

Though no clear-cut associations between hypertension and environmental or behavioral factors were identified in the study, the association with abdominal obesity and multiparity suggests that interventions aimed at lifestyle modifications to reduce these risk factors could be also useful in the prevention of hypertension.

## 1. Introduction

Noncommunicable diseases account for almost 71% of deaths throughout the world [1]. Hypertension (HTN) is a chronic disorder characterized by a persistently raised pressure in blood vessels. Globally, HTN is a major public health problem as it is the leading cause of cardiovascular diseases and is responsible for 9.4 million deaths each year in the world [2]. The prevalence of HTN is the highest in the African continent where it affects 46% of adults aged 25 y and over.

The emergence of HTN could be attributed to demographic growth and population ageing, rising urbanization, inadequate health care systems, and lifestyle changes [3–5]. Within these risk factors, poor diet is the major driver for noncommunicable diseases [6]. Hence, adherence to healthy dietary patterns is an important component for the prevention of HTN. The Dietary Approaches to Stop Hypertension (DASH) emphasized fruits, vegetables, nuts, whole grains, and low-fat dairy products and is based on lowering the consumption of sugary and sweetened beverages, sweets, and red and processed meat [7, 8]. The consumption of DASH dietary pattern was found to be protective against cardiovascular diseases, to reduce coronary heart diseases and diabetes incidence with a risk reduction of about 20% [9]. Besides, in a recent meta-analysis made by Soltani et al. (2020), it was reported that an increase of adherence to DASH patterns by an increment of 5 points reduces the risk of mortality by cardiovascular diseases, stroke, and cancer [10].

Tunisia is a Middle East and North African (MENA) country with eleven million inhabitants, located between Algeria in the east and Libya in the south. The high level of development (91^st^ out of 189 nations in 2019 on the Human Development Index) [11] is unevenly distributed, higher in the main cities and the eastern coastal regions, due to urbanization, prosperous industrial, and tourist activities. Tunisia has experienced during the last three decades a nutrition transition characterized by the emergence of overweight and obesity among all age classes [12]. A significant gender difference was found, with a higher prevalence of overweight among adult women [13]. Consistently, a diet westernization occurred characterized by the emergence of fast food and high energy and poor nutrient food consumption [14]. Consequently, chronic noncommunicable diseases progressed among younger adults, as, for example, diabetes increased from 8.6% among adult women (35–44 y.) in 1996 to 11.8 in 2005 [15].

This study is aimed at assessing the prevalence, awareness, and treatment of HTN among childbearing age Tunisian women and its association with the sociodemographic, anthropometric, and lifestyle factors. The accordance of hypertensive and nonhypertensive women to Dietary Approaches to Stop Hypertension (DASH) diet guidelines was also evaluated to have an idea on the impact of DASH diet on HTN in Tunisia [7, 16–18].

## 2. Methods

### 2.1. Setting

Tunisia is an upper-middle-income North African country (Human Development Index in 2010 was 0.683). The survey was carried out in the Great Tunis region, which is formed by four governorates, namely, Tunis, Ariana, Ben Arous, and Manouba. The Greater Tunis is mainly urban area where is living 2.5 million inhabitants in 2010 (92% live in urban settings).

### 2.2. Subjects and Study Design

The target population for the present study was all women aged between 20 and 49 y nonpregnant and living in Greater Tunis. The only exclusion criterion was pregnancy. In the framework of the Obe-Maghreb research project [19, 20], a cross-sectional survey was carried out from March 2009 to January 2010 using a two-stage random cluster sampling design of households [21].

#### 2.2.1. Sample Size Calculation

The calculation of sample size was carried out by the National Institute of Statistics. As the statistical unit for the survey was the household, the calculation of the sample size of households (*n*) with women aged 20-49 y was done using the following formula: *n* = *t*^2^ × *p* × (1 − *p*) × *DEFF* × (1/*d*^2^).

The calculation of the sample was based on the prevalence of double burden obesity and anaemia (*P* = 11.8%) among females aged 20 to 49 y. By setting a precision (*d*) of 3%, a margin of error of 5%, a cluster effect (DEFF) of 3, and a refusal rate of 5% and knowing that the population of women aged 20 to 49 y represented about 23% of the total population (according to the census of 2004) and that the average household size is equal to 4.53, the number of households required would be equal to 1520.

#### 2.2.2. Sampling and Recruitment Procedure

Households were selected using a stratified two-stage random sampling: stratification was according to the 4 governorates. In each stratum, at the first sampling stage, primary units (referred to as census districts) were selected with a probability based on the census district's size and with 76 districts in all. At the second stage, in each district, 20 households were randomly selected, and all members aged 6 months to 49 years were included in the sample. We used the subsample of 20-49 y nonpregnant women.

### 2.3. Data Collection and Measurements

#### 2.3.1. Blood Pressure

Blood pressure (BP) was measured twice with calibrated sphygmomanometers (Vaquez Laubry type, Spengler, France). The estimation of BP was based on the average of the two readings. HTN was defined as average systolic BP (SBP) and/or diastolic (DBP) levels = 140/90 mmHg [19].

#### 2.3.2. Dietary Intake and DASH Score

Dietary intake was assessed using a 3-day food record (two weekdays and one weekend day) to collect the types and amounts of the meals, foods, and beverages consumed [20]. The first step of the 3-day food record was self-administered by the subjects at home. In the day of the survey, trained dieticians reviewed unclear descriptions, errors, or omissions in the filled preprinted form and checked them with participants. For each dish, a list of ingredients, the estimated weight of the raw edible portion, and the method of preparation were collected. The accuracy of portion size of consumed foods was checked using household measures, photos of food portions [21], and known weight/specific portions.

To calculate the DASH score, food items were regrouped into 10 food groups: total grain; vegetables; fruits; dairy products; meats, poultry, and fish; nuts, seeds, dry beans, and sweets [22]. These components were chosen to achieve the DASH diet target, which is the reduction of blood pressure with a high intake of fruits, vegetables, legumes, seeds, and low-fat dairy products and whole grains and a low intake of sodium, total and saturated fats, cholesterol, and added sugars [16, 17, 22–24]. The score of each component varies from 0 to1 and the DASH accordance score is obtained by adding scores of the ten nutrient components. Then, a total nonaccordance to the DASH diet guidelines is represented by a score of 0 while a score of 10 indicates a total accordance to DASH guidelines. This scoring system was developed previously [23].

The nutritional profile of the recipes was calculated by applying to the edible part raw ingredients, yield factors to account for the change in weight during cooking, and retention factors to account for changes in their nutritional content [25]. The Tunisian food composition table [26], supplemented by the USDA table [27], additional laboratory analyses, and the Food Processor software, version 8.3 [28], were used to estimate the energy and nutritional content (macro- and micronutrients) of the consumed food items. We defined implausible energy intakes as <500 or >3500 kcal/d for women [29]. No case had been excluded.

#### 2.3.3. Covariates

Data on the level of education, profession, medical history, parity, and household socioeconomic status were collected by trained field agents using a standardized measurement protocol and questionnaire.

#### 2.3.4. Anthropometry

Body weight was measured to the nearest 0.1 kg using a digital scale, and height was measured to the nearest 0.1 cm with a stadiometer. Body mass index (BMI) ( = weight/height^2^) ≥ 25 kg/m^2^ defined overweight and BMI ≥ 30 kg/m^2^ obesity [30]. To assess abdominal fat, waist circumference (WC) was measured to the nearest 0.1 cm using a nonelastic metric measuring tape. WC ≥ 80 cm defined “increased risk abdominal obesity” and WC ≥ 88 cm “high risk abdominal obesity” [30].

#### 2.3.5. Tobacco

Current smokers of cigarettes were those who had smoked in the past one month (cigarettes, chichi, *Neffa*).

#### 2.3.6. Sports

Physical activity of subjects during the 7 days preceding the survey was assessed using a validated frequency questionnaire [31]. Sport activities were scored “yes” for women who regularly carry out a sport activity and “no” for women who do not exercise at all any sport.

### 2.4. Data Management and Statistical Analysis

Data entry screens including quality checks as well as validation by double-entry used EpiData Software version 3.1 (The Epidata Association, Odense, Denmark, 2008). Data management was performed using the Stata software version13.0 (Stata Corporation, College Station, Texas, 2013) software. Results are shown as mean ± standard error. The association between categorical variables was evaluated by the test of chi-square. The odds ratio (OR) of being hypertensive in association with covariates was assessed using logistic regression. To control for potential confounding due factors, we adjusted for the socioeconomic and demographic characteristics after the selection of an appropriate reference category. For testing and confidence intervals, alpha 5% threshold was chosen.

### 2.5. Ethical Consideration

The Ethical Committee of the Tunisian National Institute of Nutrition and Food Technology and the Tunisian National Council of Statistics approved the survey protocol (visa n°2/2009). After being informed about the purpose and procedures of the survey, all participants gave their consent to take part in the study, which was held at primary health-care centers and hospitals. This study is also registered in the ClinicalTrials.gov registry. The Identifier number is NCT01844349.

## 3. Results

### 3.1. Sample Characteristics

Of the 1887 eligible nonpregnancy women aged 20-49 y, 1689 were involved in the analyses, yielding an overall response rate of 89.5%. The mean age women included in the study was 36.1 ± 0.3 y and mean BMI 28.3 ± 0.2 kg/m^2^. Two-thirds of women were married, and more than one-third had no children. Only one-tenth of women had never attended school, and one-third worked outside the home.

### 3.2. Prevalence of HTN

Table 1 provides the estimated prevalence and adjusted associations of HTN by sociodemographic characteristics and lifestyle factors. Overall, 21.4% of childbearing age Tunisian women were hypertensive. The prevalence of HTN increased significantly with age, BMI, and WC. HTN was more prevalent (*P* < 0.0001) among married women (25.9%) in comparison with single, divorced, or widowed ones (12.3%). A greater prevalence of HTN was observed in women with 3 children or more (30.0%) as compared with women without children (10.9%) or with one or two children (21.4%). Menopausal women were more hypertensive than premenopausal (40.2% vs. 25.0%; *P* = 0.0011). The prevalence of HTN was higher among illiterate group compared to intermediate or higher education level groups. The area of living as well as the household size and the economic level had no influence on the prevalence of HTN in women. As regards lifestyle factors, no significant differences in smoking habits and physical activity were noticed (Table 1). HTN was more frequent for women who reported a family history of HTN. Among the total hypertensive subjects, 29.6% were aware of their situation, and almost the fifth was on antihypertensive treatment. Sixty-five percent of hypertensive women, who were aware of their condition, were receiving treatment.

After adjustment for all socioeconomic, anthropometric, and behavioral covariates, the prevalence of HTN increased markedly with age (OR = 2.90 [1.25‐7.50]; *P* = 0.034), obesity (OR = 1.61 [1.15‐3.06]; *P* = 0.014), high-risk abdominal obesity (OR = 2.76 [1.47‐5.18]; *P* = 0.002), parity (OR = 2.02 [1.09‐3.76]; *P* = 0.027), and family history of HTN (OR = 1.77 [1.24‐2.53]; *P* = 0.002).

### 3.3. DASH Score

Table 2 presents a comparison of the accordance to DASH diet guidelines between hypertensive and nonhypertensive women. Significant differences were observed for total grain, sweets, and sodium intake. Hypertensive women achieved the highest score for total grain intake (81.6% of hypertensive women vs. 71.5% of nonhypertensive women; *P* = 0.041), whereas nonhypertensive women had better adherence to the DASH diet guidelines for sweets and sodium intake. The mean DASH accordance score, obtained after the addition of the different component scores, was 4.93 for hypertensive women and 4.86 for nonhypertensive women (*P* = 0.0556).

## 4. Discussion

In the present study, we found significant associations between HTN and age, BMI, WC, parity, and family history of HTN. After considering potential confounders, these significant associations were also present except for WC factor. Besides, the adherence to DASH diet score was elevated for both hypertensive and nonhypertensive women but no significant associations between DASH diet food groups and HTN were found.

Our study showed that approximately one Tunisian woman out of five was hypertensive. This is lower than the prevalence of 36.1% previously reported by Allal-Elasmi et al. (2012) [32] in the same sampling area. The difference in the age groups of both studied populations (35-69 y [32] vs. 25-49 y in our study) could explain such dissimilarity in HTN rates. Age plays an important role in increasing blood pressure, and people aged 50 y and over have markedly high risks of cardiovascular disease (CVD) due to HTN [33–35]. This hypothesis is confirmed by previous studies on Tunisian population, where the prevalence of HTN was 4.7% among adolescent [36] and 55.5% among elderly women [37]. Compared to studies conducted in other African countries, with an overall mean age close to ours, the prevalence of HTN among women is similar to those reported in Chad (21.8%) and Uganda (21.7%) countries [38, 39]. Higher amounts were recorded from Ghana (28.1%) [40] and Togo (27.6%) nations [41]. Generally, the prevalence of HTN among Tunisian women is within the range of [18.7%-36.4%] recorded in Middle East and North Africa (MENA) region, representing the lowest estimates of HTN in middle- and low-income countries in the world [4].

In our survey, almost thirty percent of hypertensive women were aware of their condition. Higher rates were previously reported from adults (44.8% and 41.0%) and elderly (81.0%) living in Tunisian country [37, 42, 43]. The mean age of subjects, which was the lowest in our study, could be the main cause of variability in HTN awareness. Treatment of HTN was achieved by almost two-thirds of women who were aware of their hypertensive status. This relatively elevated rate could be attributed to the availability of free government-supplied antihypertensive drugs distributed in Tunisian hospitals.

In this study, obesity (BMI above 30 kg/m^2^) and abdominal obesity (WC above 102 cm) were among the most influencing factors on blood pressure in Tunisian women. Moreover, salt consumption was significantly higher among hypertensive women. These results suggest that caloric intake but also nutrient composition, especially sodium content, may have an influence on the evolution of blood pressure.

Many mechanisms have been proposed to explain the correlation between obesity and HTN. These mechanisms are largely influenced by the type of adipose tissue and its distribution. Thus, subjects with morbid obesity present an amplified risk of HTN compared to overweight or normal weight persons. At the same time, any loss of weight results in a regression of blood pressure, even in normotensive obese [44]. The association between WC and HTN has been accorded to hyperinsulinemia due to excess abdominal fat [45]. As regards salt intake, there is strong evidence suggesting that a high-salt diet is a major factor increasing blood pressure [46–48]. Large amounts of salt reduce the kidney's ability to evacuate sodium and therefore cause a rise in blood pressure [49]. Furthermore, salt has other harmful effects, independent of and sometimes additive to its influence on blood pressure, such as its association with obesity through soft drink intake [50]. Our findings are in line with those reported by Bruno et al. (2016) [51], who stated that body weight and dietary habits are the main factors influencing blood pressure in a cohort of Italian young adults. Other national investigations, conducted in Tunisia or elsewhere, reported comparable findings [36, 43, 52, 53].

Parity has a strong positive effect on HTN as well as family history of HTN. These results are consistent with those reported in previous research studies. According to Taylor et al. (2008), the number of pregnancies and the parental history of HTN may influence the risk for developing HTN during lifetime [54]. Moreover, the same authors found a statistically significant difference between the number of children and systolic blood pressure in West African Dogon Women [55]. Giubertoni et al. (2013) suggested that women with at least one child had almost threefold risk of being hypertensive in comparison with nulliparous women [56]. This correlation between parity and HTN was attributed to physiological changes in blood perfusion during pregnancy, including increases in cardiac output and blood volume and decreases in systemic and pulmonary vascular resistance [57]. Pregnancy-related weight gain could also be associated with increases in blood pressure [54, 56]. As regards family history, it is well established that this factor is a strong predictor of HTN contributing to about 30% of blood pressure variance [58]. Then, studying family history of HTN may be an important strategy for the prevention and control of HTN.

In the present study, at least 60% of childbearing age Tunisian women met the DASH diet guidelines for total grain, meats, nuts, fats, and sweets. However, the consumption of vegetables, fruits, dairy products, and sodium did not comply with DASH diet pattern. The mean DASH diet accordance score of Tunisian women was relatively high compared to data recorded elsewhere [24, 59]. This is probably due to the dietary habits of Tunisian population. Tunisia is a typical country of the MENA region that is undergoing a nutritional and epidemiological transition. This transition was accompanied with a change in food consumption and trade by an increase of cereals, oils, and sugar importation [60]. Intake of vegetables and fruits has also increased leading to the improvement of food diversification in Tunisia. No statistical differences were observed between hypertensive and nonhypertensive women on the adherence to the DASH diet guidelines. However, hypertensive women had a significantly higher intake of total grains, sodium, and sweets than nonhypertensive women. Consequently, they were less likely to follow a healthy diet compared to those without HTN diagnosis. Results on multivariable regression analysis showed no association between HTN and DASH diet food groups. This is consistent with the findings of Harrington et al. (2013), who examined the relationship between individual DASH components and systolic blood pressure in older Irish men and women and did not found statistical associations except for low fat milk [17].

## 5. Conclusion

In this study, almost one Tunisian woman out of five was hypertensive. Age, family history, parity, abdominal fat, and BMI status were found as risk factors for HTN. Adherence to DASH diet score by Tunisian women was relatively high compared to data recorded elsewhere and similar between hypertensive and nonhypertensive subjects. The multivariable regression analysis revealed no relationships between HTN and DASH diet food groups. This study, carried out during the period of nutrition transition that occurred in Tunisia, provides a baseline for implemented governmental policies to tackle chronic noncommunicable disease.

## Figures and Tables

**Table 1:** Prevalence and adjusted odds ratio of HTN by sociodemographic characteristics, body mass index, waist circumference, and lifestyle factors.

Variables	*N*	HTN			
		%^1^	*P* ^2^	Adjusted OR^3^	*P* ^4^
				(CI 95%)	
Age (year)					
*20-29*	509	8.7	<0.0001	1 (ref)	
*30-39*	496	18.1		0.45 (0.02-8.64)	0.60
*40-49*	684	33.0		2.90 (1.25-7.50)	0.034
Body mass index (kg/m^2^)					
*<25*	524	8.0	<0.0001	1 (ref)	
*25-29*	555	17.9		0.92 (0.51-1.68)	0.79
*≥30*	610	35.5		1.61 (1.15-3.06)	0.014
Waist circumference (cm)					
<88	487	7.6	<0.0001	1 (ref)	
88-102	391	13.4		1.27 (0.72-2.23)	0.40
≥102	811	33.3		2.76 (1.47-5.18)	0.002
Marital status					
*Single, divorced, or widowed*	608	12.3	<0.0001	1 (ref)	
*Married*	1081	25.9		0.77 (0.46-1.27)	0.30
Parity					
*0*	621	10.9	<0.0001	1 (ref)	
*1 or 2*	495	21.4		1.60 (0.83-3.07)	0.15
*3 and more*	573	30.0		2.02 (1.09-3.76)	0.027
Menopause					
*No*	1552	21.4	0.0011	1 (ref)	
*Yes*	137	40.2		1.49 (0.97-2.29)	0.067
Level of instruction					
*Secondary and graduate*	894	16.9	0.0001	1 (ref)	
*Primary*	613	25.2		1.10 (0.64-1.88)	0.73
*No schooling*	182	30.6		1.02 (0.72-1.43)	0.93
Professional activity					
*Yes*	564	17.8	0.014	1 (ref)	
*No*	1125	23.1		1.29 (0.93-1.80)	0.13
Area of living					
*Rural*	214	15.6	0.22	1 (ref)	
*Urban*	1474	21.8		1.68 (0.81-3.46)	0.16
Household size					
1-4	687	22	0.91	1 (ref)	
5	480	21.1		0.69 (0.43-1.10)	0.11
6 and +	522	21.1		0.71 (0.46-1.10)	0.13
Household economic level					
*High*	576	22.2	0.53	1 (ref)	
*Middle*	553	22.4		1.37 (0.87-2.15)	0.17
*Low*	560	19.6		1.22 (0.80-1.85)	0.34
Sport activity					
*Yes*	114	21.2	0.62	1 (ref)	
*No*	1575	23.3		1 (0.22-1.01)	0.056
Smoking tobacco					
*Nonsmoker*	1568	21.9	0.27	1 (ref)	
*Current smoker*	121	13.0		0.53 (0.26-1.08)	0.079
Family history of HTN					
*No*	1333	16.1	0.0019	1 (ref)	
*Yes*	355	24.3		1.77 (1.24-2.53)	0.0020

^1^Values are percentages based on weighted data. ^2^*F*-test for continuous variables, chi-square test for categorical variables (design-based tests). ^3^OR: value for odds-ratio (95% CIs), adjusted for age and all the variables in the first column. ^4^*P* value for logistic regression models accounting for survey design among categories of variable. After adjustment for age, energy intake, and all nutritional covariates, no associations were observed between HTN and DASH diet components.

**Table 2:** Distribution and scoring of the DASH diet index among hypertensive and nonhypertensive women.

Dash guideline	Score	Hypertensive (*N* = 349)		Nonhypertensive (*N* = 1340)		*P* value
		*N*	%	*N*	%	
Total grain						
≥7 servings/day	1	285	81.6	955	71.5	**0.0078**
5–6 servings/day	0.5	50	14.6	283	21.7	
<5 servings/day	0	14	03.8	102	06.8	
Vegetables						
≥4 servings/day	1	26	06.4	75	05.6	0.0544
2–3 servings/day	0.5	138	40.2	463	33.5	
<2 servings/day	0	185	53.4	802	60.9	
Fruits						
≥4 servings/day	1	13	03.4	58	04.7	0.4417
2–3 servings/day	0.5	55	15.9	191	13.9	
<2 servings/day	0	281	80.7	1091	81.5	
Dairy foods						
≥2 servings/day	1	11	03.2	61	04.9	0.4266
1 serving/day	0.5	50	13.5	193	13.9	
<1 serving/day	0	288	83.4	1086	81.5	
Meats, poultry, and fish						
≤2 servings/day	1	343	98.6	1308	97.6	0.3914
3 servings/day	0.5	0	00.0	1	00.0	
≥4 servings/day	0	6	01.4	31	02.4	
Nuts, seeds, and dry beans						
≥4 servings/week	1	226	66.0	818	61.0	0.2653
2–3 servings/week	0.5	110	30.3	456	34.1	
<2 servings/week	0	13	03.7	66	04.8	
% kcal from fat						
≤30%	1	258	73.6	963	71.5	0.8004
31%–32%	0.5	30	08.8	125	09.5	
≥33% 0	0	61	17.6	252	18.9	
% kcal from saturated fatty acids						
≤10%	1	333	95.2	1240	92.2	0.1908
11%–12%	0.5	7	02.1	49	03.6	
≥13%	0	9	02.7	51	04.2	
Sweets						
≤5 servings/week	1	241	68.8	1028	77.0	**0.0120**
6–7 servings/week	0.5	66	19.2	176	13.4	
≥8 servings/week	0	42	12.1	136	09.6	
Sodium						
≤1500 mg/day	1	1	00.3	2	00.2	**0.0238**
1501–2400 mg/day	0.5	19	05.4	142	11.2	
≥2401 mg/day	0	329	94.3	1196	88.7	

## Data Availability

Data are available from the corresponding author upon a reasonable request.
